# A functionally distinct neutrophil landscape in severe COVID-19 reveals opportunities for adjunctive therapies

**DOI:** 10.1172/jci.insight.152291

**Published:** 2022-01-25

**Authors:** Rachita Panda, Fernanda V.S. Castanheira, Jared M. Schlechte, Bas G.J. Surewaard, Hanjoo Brian Shim, Amanda Z. Zucoloto, Zdenka Slavikova, Bryan G. Yipp, Paul Kubes, Braedon McDonald

**Affiliations:** 1 Snyder Institute for Chronic Diseases,; 2 Department of Physiology and Pharmacology,; 3 Department of Critical Care Medicine, and; 4 Department of Microbiology, Immunology, and Infectious Diseases, Cummings School of Medicine, University of Calgary, Calgary, Alberta, Canada.

**Keywords:** Immunology, Infectious disease, Innate immunity, Neutrophils

## Abstract

Acute respiratory distress syndrome (ARDS) is a life-threatening syndrome, constituted by respiratory failure and diffuse alveolar damage that results from dysregulated local and systemic immune activation, causing pulmonary vascular, parenchymal, and alveolar damage. SARS-CoV-2 infection has become the dominant cause of ARDS worldwide, and emerging evidence implicates neutrophils and their cytotoxic arsenal of effector functions as central drivers of immune-mediated lung injury in COVID-19 ARDS. However, key outstanding questions are whether COVID-19 drives a unique program of neutrophil activation or effector functions that contribute to the severe pathogenesis of this pandemic illness and whether this unique neutrophil response can be targeted to attenuate disease. Using a combination of high-dimensional single-cell analysis and ex vivo functional assays of neutrophils from patients with COVID-19 ARDS, compared with those with non-COVID ARDS (caused by bacterial pneumonia), we identified a functionally distinct landscape of neutrophil activation in COVID-19 ARDS that was intrinsically programmed during SARS-CoV-2 infection. Furthermore, neutrophils in COVID-19 ARDS were functionally primed to produce high amounts of neutrophil extracellular traps. Surprisingly, this unique pathological program of neutrophil priming escaped conventional therapy with dexamethasone, thereby revealing a promising target for adjunctive immunotherapy in severe COVID-19.

## Graphical abstract

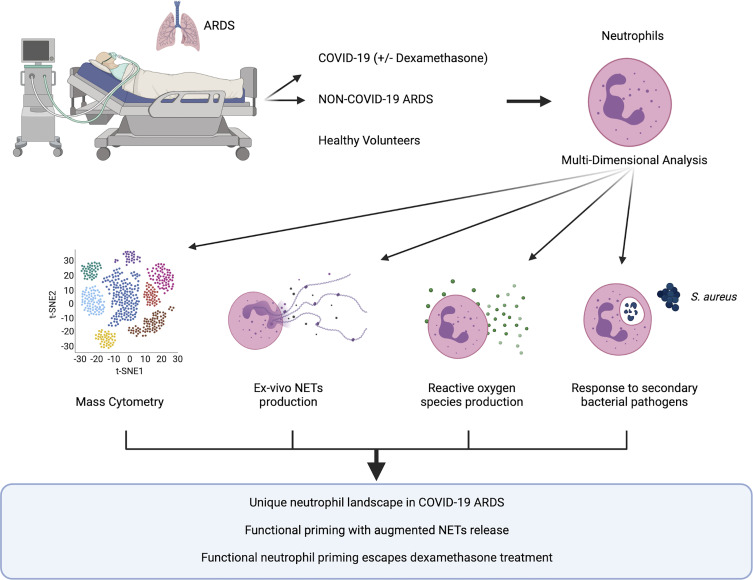

## Introduction

Acute respiratory distress syndrome (ARDS) is a life-threatening syndrome, constituted by hypoxemic respiratory failure and diffuse alveolar damage that results from an aberrant host response to respiratory and systemic insults, with the most common cause being severe pneumonia (1). Dysregulated local and systemic inflammation triggers widespread pulmonary vascular, parenchymal, and alveolar damage, resulting in failure of gas exchange and critical illness (2). The mainstay of treatment for ARDS is supportive care, including the use of mechanical ventilation, but ongoing research efforts strive to define the mechanisms of pathogenesis to enable targeted therapeutic interventions (2).

SARS-CoV-2 infection has emerged as the dominant cause of ARDS worldwide in the context of the COVID-19 pandemic (3). In response, there has been a vast increase in research attempting to uncover why SARS-CoV-2 infection is so prone to causing ARDS, and much has been learned about the pathogenesis, including aberrant local and systemic immune responses elicited by the virus. Transcriptomic and proteomic analyses of the immune landscape in the bloodstream as well as lung tissue have revealed that COVID-19 is associated with a dysregulated myeloid cellular response and, in particular, that the progression toward severe disease is heralded by aberrant neutrophil activation (4–8). Autopsy studies of lung tissue samples from patients who died of COVID-19 have shown evidence of neutrophil infiltration within the pulmonary microvasculature and alveoli, albeit to a lesser extent than that seen with ARDS caused by bacterial pneumonia, suggesting distinct mechanisms of neutrophil responses to SARS-CoV-2 infection compared with other causes of ARDS (9, 10). Furthermore, neutrophils within the pulmonary microvasculature of severe COVID-19 are associated with widespread neutrophil extracellular trap (NETs) production (11–14). This dysregulated neutrophil effector response is thought to contribute to impaired gas exchange through the induction of microvascular thrombosis and perfusion defects, as well as tissue injury and diffuse alveolar damage.

Given the growing appreciation that immune-mediated pathology is the primary driver of disease in COVID-19 ARDS, it has become the focus of therapeutic intervention. Currently, the most established immunomodulatory therapy for severe COVID-19 is dexamethasone, which has been shown to reduce mortality in moderate-to-severe disease (15). However, the mechanisms by which dexamethasone improves clinical outcomes in COVID-19 are incompletely understood, and therefore, opportunities may exist for adjunctive immunomodulation directed at mechanisms that escape the activity of dexamethasone.

Key outstanding questions are whether COVID-19 ARDS drives a unique program of neutrophil maturation, activation, or effector functions that contributes to the severe pathogenesis of this pandemic illness and whether this unique neutrophil response could be targeted therapeutically to supplement established therapies such as dexamethasone. To address these questions, we used a combination of high-dimensional single-cell analysis and ex vivo functional assays of neutrophils from patients with COVID-19 ARDS, compared with patients with non-COVID ARDS (caused by bacterial pneumonia). We observed that COVID-19 ARDS programs a distinct landscape of neutrophil activation compared with non-COVID ARDS and that neutrophils from patients with COVID-19 are uniquely primed to produce NETs. This functional priming was an intrinsically programmed response during SARS-CoV-2 infection, as it could not be induced in healthy neutrophils by incubation with the plasma of patients with COVID-19, and neutrophils remained competent to respond to secondary bacterial challenges. Consistent with data in previous reports, we observed a trend toward worse clinical outcomes in patients with high levels of NET production, but surprisingly, we found that this was not modified in patients receiving dexamethasone treatment. Together, these data reveal a unique pathological program of neutrophil priming in COVID-19 that escapes conventional therapy and, therefore, may represent a promising target for adjunctive treatments that provide synergistic benefits in the fight against severe COVID-19.

## Results

### The systemic immune response in COVID-19 ARDS is dominated by a unique neutrophil landscape and is distinct from non-COVID ARDS.

**Figure 1:**
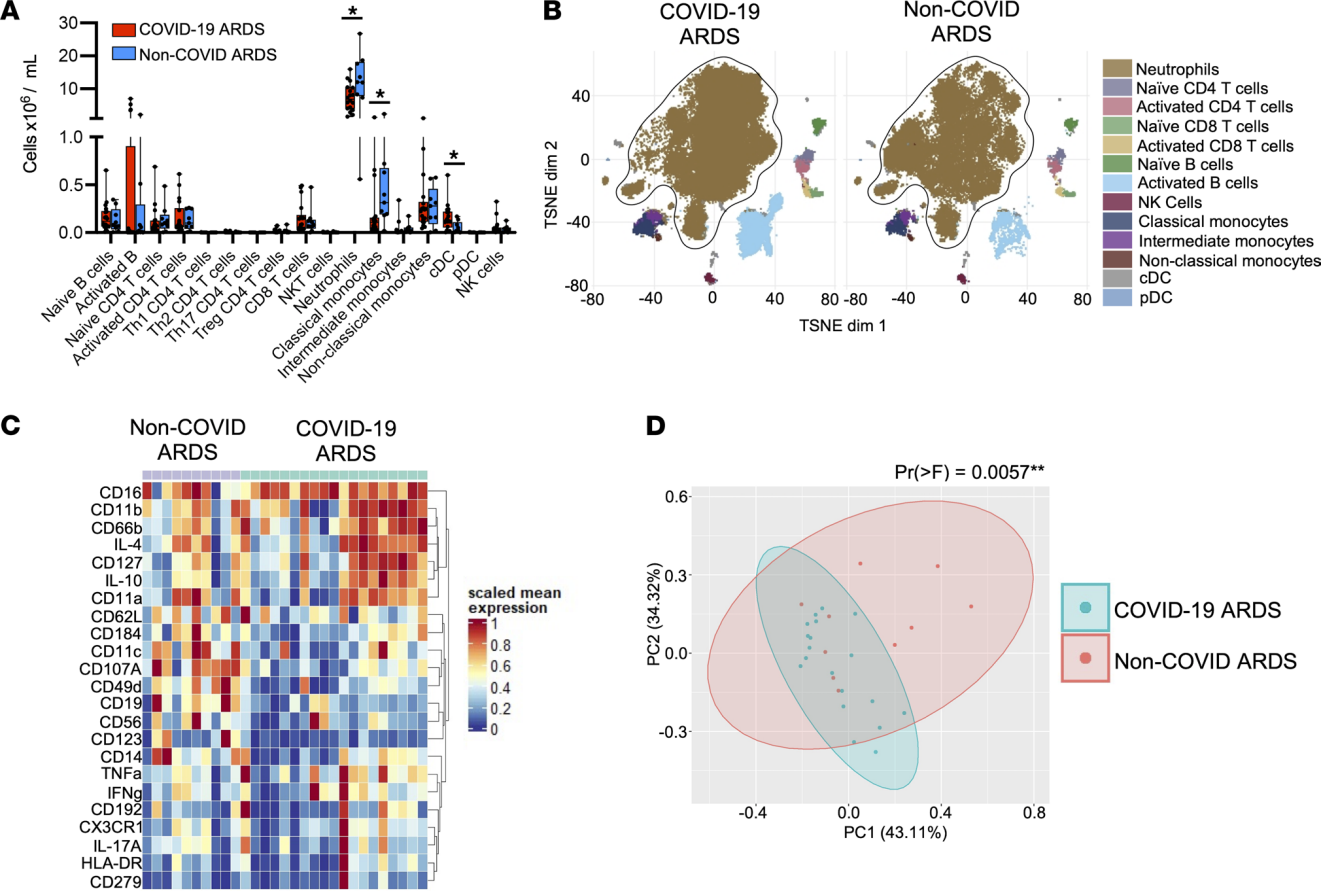
A distinct neutrophil landscape dominates the systemic immune response in COVID-19 ARDS. (**A**) Abundance of immune cell populations in the blood of patients with COVID-19 ARDS (*n* = 19) compared with non-COVID ARDS (*n* = 10) at ICU admission. Data are shown as the median ± range; *P < 0.05 by Mann-Whitney *U* test. (**B**) Dimensionality reduction using tSNE of single-cell mass cytometry data demonstrating clustering of major immune cell populations in the blood of patients on day 1 of ICU admission. (**C**) Expression levels of selected neutrophil surface and intracellular markers, and (**D**) principal component analysis of neutrophil marker expression at ICU admission in patients with COVID-19 ARDS and non-COVID ARDS. **P < 0.01 by permutational multivariate ANOVA test.

It is now well established that SARS-CoV-2 induces a unique systemic immune response that is central to the pathogenesis of severe COVID-19, including a putative role for neutrophils in the progression of disease during SARS-CoV-2 infection. To systematically examine the contribution of neutrophils to systemic immune dysregulation in severe COVID-19, we investigated the cellular immune landscape in the bloodstream of patients with ARDS caused by COVID-19 pneumonia or bacterial pneumonia (hereafter, non-COVID ARDS). Patients were balanced with respect to age, sex, illness severity, and treatments including mechanical ventilation (Table 1). Patients were enrolled prospectively, and blood specimens were collected on admission to the intensive care unit (ICU) and again on day 7 in survivors who remained in the ICU. We performed high-dimensional single-cell analysis using mass cytometry on whole blood from patients with COVID-19 ARDS, compared with that from those with non-COVID ARDS. Neutrophils were the dominant immune cell in the blood of all patients, with the total quantity of neutrophils being higher in patients with non-COVID ARDS (Figure 1A). Comparatively small differences were seen in other immune cell populations, such as classical monocytes and conventional dendritic cells (Figure 1A). In addition to quantitative difference, we next investigated whether the neutrophils differed phenotypically between patients with COVID-19 and patients with non-COVID ARDS. Dimensionality reduction using tSNE revealed marked changes in the clustering of neutrophils between patients with COVID-19 and patients with non-COVID ARDS, whereas clusters of other major cell populations (T cells, B cells, monocytes, etc.) were largely stable (Figure 1B). To further investigate this difference in the neutrophil compartment, we analyzed expression levels of 23 neutrophil-relevant surface and intracellular markers on gated neutrophils and found significant differences driven by augmented expression of CD11b, CD66b, and CD11a and reduced CD62L and CD107A on neutrophils from patients with COVID-19 ARDS, consistent with a more activated phenotype (Figure 1, C and D). Together these data demonstrate a distinct neutrophil landscape in COVID-19 ARDS that is a dominant aspect of the systemic immune response to SARS-CoV-2.

**Figure 2:**
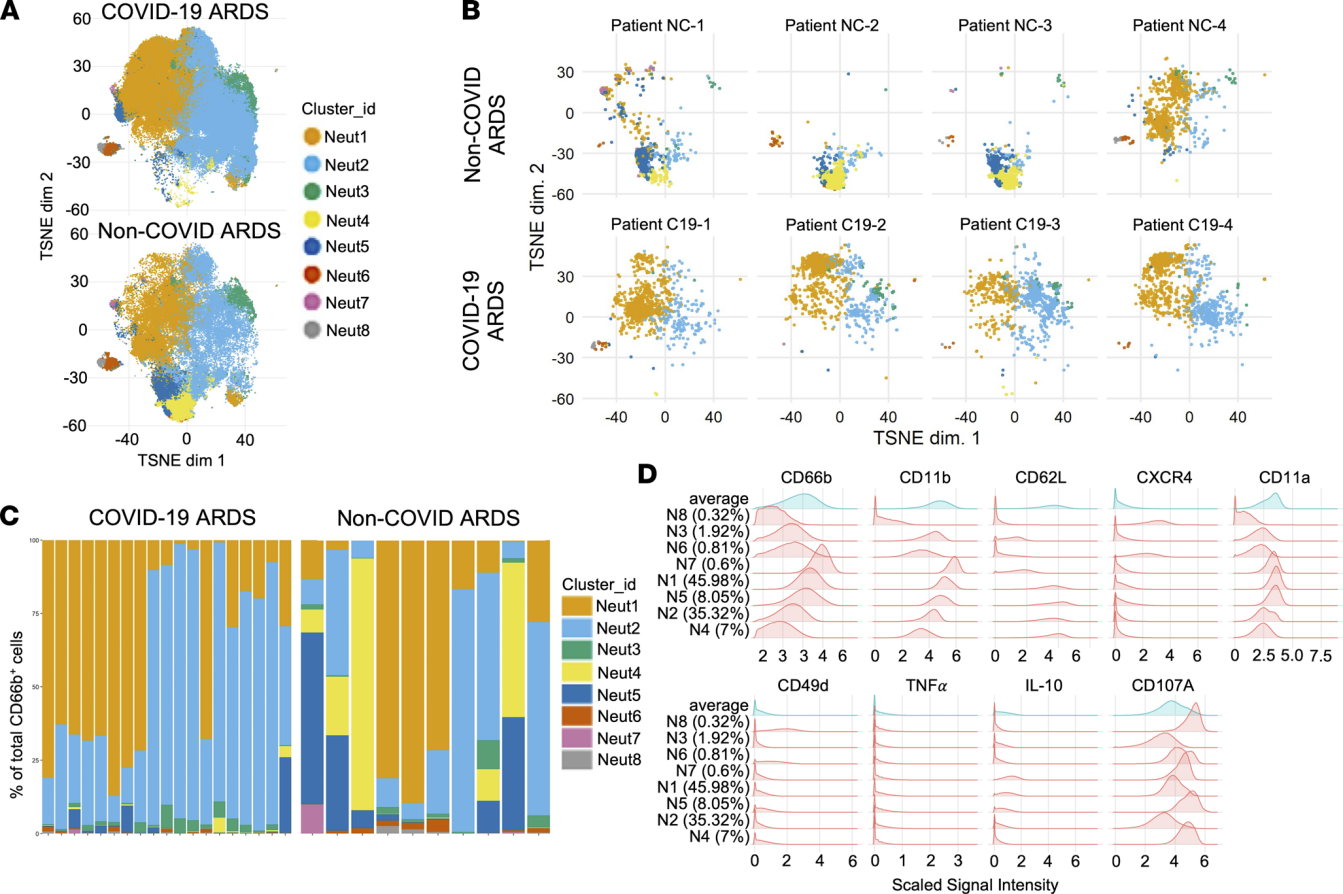
A distinct neutrophil landscape in COVID-19 ARDS versus non-COVID ARDS. Mass cytometry analysis of neutrophils showing (**A**) unsupervised clustering of neutrophil single-cell events using FlowSOM in samples from patients with COVID-19 ARDS (*n* = 19) and with non-COVID ARDS (*n* = 10) and (**B**) representative clustering of neutrophils in selected individual patients with COVID-19 ARDS and non-COVID ARDS. (**C**) Relative abundance of neutrophils clusters in individual patient samples, and (**D**) histogram plots demonstrating expression of key surface and intracellular markers that differentiate the identified neutrophil clusters.

To further resolve the differences between COVID-19 and non-COVID neutrophil responses, we performed unsupervised clustering of neutrophil single-cell events with FlowSOM and identified 8 distinct clusters of neutrophils (Figure 2A). Comparing our 2 patient populations, we identified significant differences in the presence and abundance of distinct neutrophil clusters between patients with COVID-19 and patients with non-COVID ARDS (Figure 2, A–C, and Supplemental Figure 1; supplemental material available online with this article; https://doi.org/10.1172/jci.insight.152291DS1). Again, we observed dominance by mature and activated neutrophil clusters (Neut 1 and Neut2) in all patients with COVID-19, whereas the neutrophil compartment in patients with non-COVID ARDS was more heterogeneous, with enrichment of Neut4 that displayed a more immature phenotype (Figure 2, C and D). In our cohort of non-COVID ARDS patients, we did not identify any trends in neutrophil clustering based on the infecting bacterial pathogen. Furthermore, differences in neutrophil marker expression and single-cell clustering persisted even when patients with COVID-19 ARDS receiving dexamethasone therapy were excluded (Supplemental Figure 2). Collectively, these data indicate that patients with COVID-19 ARDS display a unique systemic immune response that is dominated by a distinct neutrophil landscape compared with patients with non-COVID ARDS and is driven by a shift toward a more mature and activated neutrophil phenotype.

### The unique neutrophil landscape in COVID-19 ARDS is associated with functional priming.

**Figure 3:**
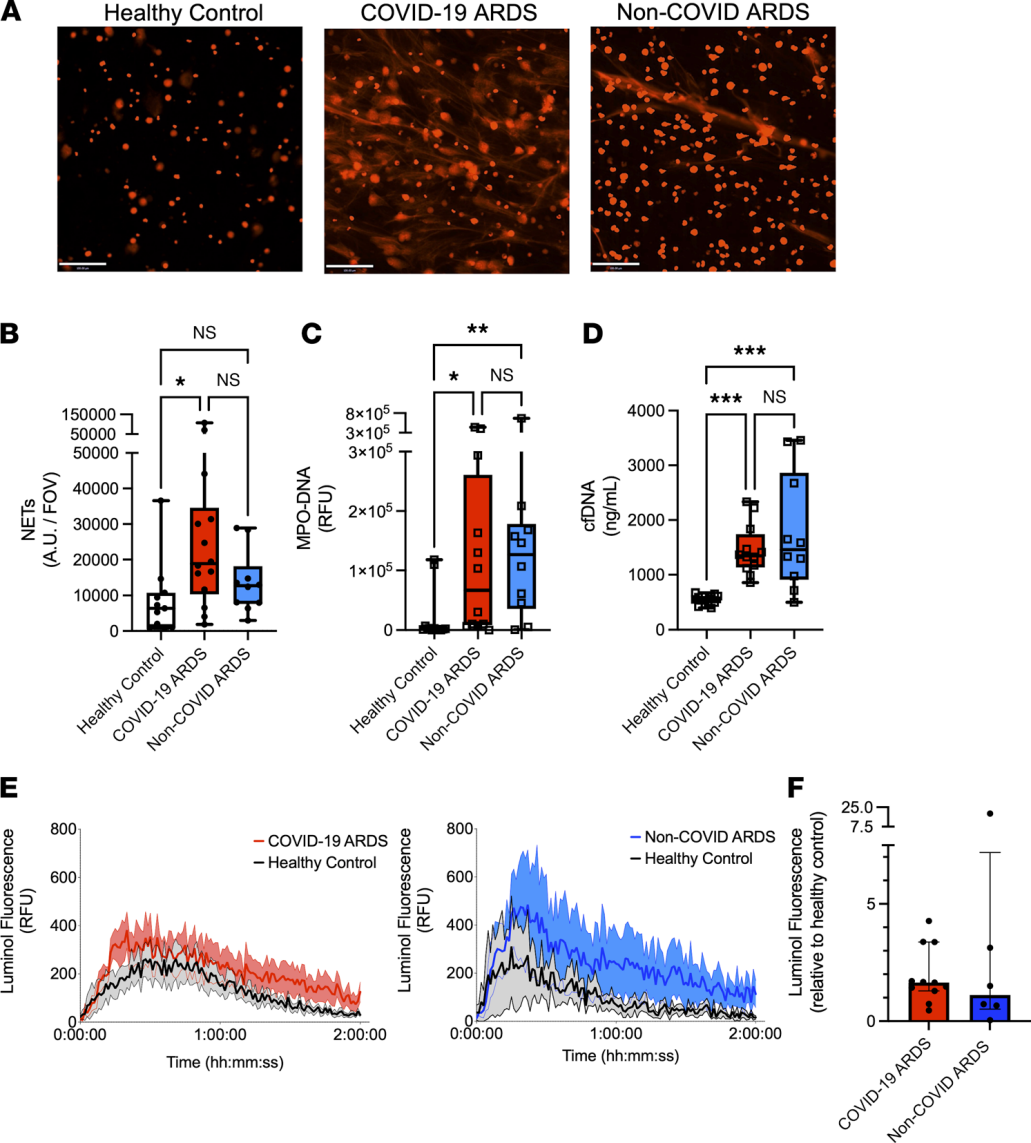
Neutrophils in COVID-19 ARDS are uniquely primed to produce neutrophil extracellular traps. (**A**) Representative images of neutrophil extracellular trap (NET) release from healthy control neutrophils (left), neutrophils from patients with COVID-19 ARDS (center), and neutrophils from patients with non-COVID ARDS (right) (representative examples of data in **B**). Scale bars: 100 μm. (**B**) Quantitation of area per field of view covered by NETs released from neutrophils ex vivo from patients with COVID-19 (*n* = 14) and non-COVID ARDS (*n* = 10) at ICU admission as well as healthy controls (*n* = 11). Dots represent individual patients, bars represent median, and whiskers represent the range; *P < 0.05 by Kruskal-Wallis test with post hoc Dunn’s test. (**C** and **D**) Plasma levels of (**C**) MPO-DNA complexes and (**D**) cell-free DNA (cfDNA) in patients with COVID-19 (*n* = 12) and non-COVID ARDS (*n* = 10) at ICU admission as well as healthy controls (*n* = 13). Dots represent individual patients, bars represent median, and whiskers represent the range; *P < 0.05 **P < 0.01 ***P < 0.001 by Kruskal-Wallis test with post hoc Dunn’s test. (**E** and **F**) ROS production by neutrophils detected by luminol fluorescence assay, shown as (**E**) relative fluorescence units (RFU) over time and (**F**) AUC of luminol fluorescence of neutrophils from patients with COVID-19 (*n* = 11) and non-COVID (*n* = 6) ARDS relative to healthy control neutrophils at ICU admission. Dots represent individual patients, bars represent median, and whiskers represent the range.

Given the distinct phenotypic landscape of neutrophils observed in COVID-19 ARDS, we next sought to determine whether neutrophils in COVID-19 ARDS display unique effector functionality. We performed ex vivo live-cell functional analyses on neutrophils isolated from patients with COVID-19, compared with patients with non-COVID ARDS, to quantify key effector functions, including the release of NETs and ROS. To understand the intrinsic functional activity of neutrophils in response to COVID-19 or non-COVID ARDS, we first assessed the production of NETs and ROS in otherwise unstimulated cells. Neutrophils from patients with COVID-19 ARDS at ICU admission displayed significantly higher basal NET release compared with neutrophils from healthy donors, whereas no significant difference was observed in non-COVID ARDS neutrophils (Figure 3, A and B). As expected, neutrophils from healthy volunteers did not release NETs under basal conditions (Figure 3, A and B). We next quantified the levels of circulating cell-free DNA (cfDNA) and NETs (myeloperoxidase-DNA [MPO-DNA] complexes) in patient plasma and again observed significantly elevated NETs and cfDNA in the plasma of patients with COVID-19 (Figure 3, C and D), as previously reported (16, 17). In patients with non-COVID ARDS, the levels of circulating cfDNA and MPO-DNA complexes were also elevated compared with healthy controls (Figure 3, C and D). The observation of elevated total plasma cfDNA and NETs despite blunted ex vivo NET release by neutrophils from patients with non-COVID ARDS may be due to a cumulative effect of significantly higher numbers of neutrophils in the circulation of these patients compared with those with COVID-19 ARDS (Figure 1A). Augmented NET release in COVID-19 neutrophils was sustained over time, with a trend toward increased production of NETs, as well as significantly elevated plasma cfDNA and MPO-DNA complexes on day 7 of illness (Supplemental Figure 3, A–C). Neutrophils from all groups were found to release similar levels of NETs upon stimulation with phorbol myristate acetate-13 (PMA), suggesting that the capacity to respond to maximal activation was not different (Supplemental Figure 4A). Finally, to determine whether NET production was driven by a particular subset/cluster of neutrophils, we investigated the correlation between neutrophil cluster abundance and ex vivo NET release as well as circulating NETs (MPO-DNA complexes). Although not achieving statistical significance, we observed a notable positive correlation between the Neut2 cluster abundance and higher production of NETs (Supplemental Figure 4B). In contrast to NET release, ROS production by neutrophils was not different between patients with COVID-19 and patients with non-COVID ARDS either at admission or after 7 days in the ICU (Figure 3, E and F, and Supplemental Figure 3D). Together, these data indicate that the distinct neutrophil landscape in COVID-19 ARDS is marked by functional priming of specific effector mechanisms, including NET release.

### Functional priming of neutrophils in COVID-19 ARDS is not induced by circulating inflammatory mediators.

**Figure 4:**
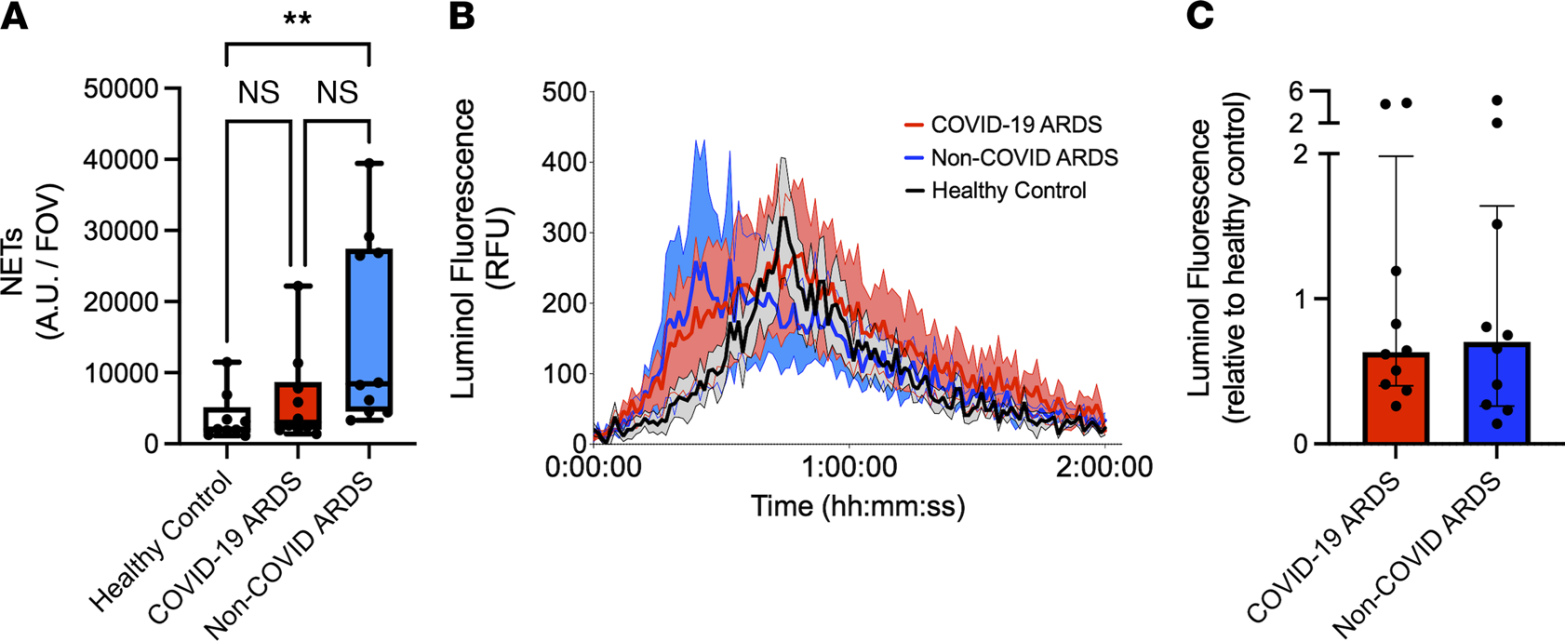
Neutrophil priming is not induced by inflammatory mediators in plasma. (**A**) Ex vivo imaging of NET release quantified as NET area per field of view from neutrophils from healthy volunteers incubated with plasma of healthy controls (*n* = 9), patients with COVID-19 ARDS (*n* = 10) and patients with non-COVID ARDS (*n* = 10). Dots represent individual patients, bars represent median, and whiskers represent the range; **P < 0.01 by Kruskal-Wallis test with post hoc Dunn’s test. (**B** and **C**) ROS production by neutrophils detected by luminol fluorescence assay shown by (**B**) relative fluorescence units (RFU) and (**C**) AUC of luminol fluorescence following stimulation of healthy donor neutrophils with plasma from patients with COVID-19 ARDS (*n* = 10) or patients with non-COVID ARDS (*n* = 10) relative to healthy control plasma. Dots represent individual patients, bars represent median, and whiskers represent the range.

Plasma biomarker analyses in various cohorts of patients with severe COVID-19 have shown a circulating “cytokine storm” that is distinct from other causes of sepsis and ARDS (18). Previous work by us and others (19, 20) has shown that circulating inflammatory mediators in patients with septic shock induce neutrophil hyperproduction of effector mechanisms, including the release of NETs. In a recent study of primarily mild and moderate COVID-19 pneumonia, circulating factors in plasma were also shown to elicit release of NETs (16). To determine whether the functional priming and activation of neutrophils observed in COVID-19 ARDS were a response to the unique circulating cytokine storm in these patients, we assessed the ability of patient plasma to induce NET release by neutrophils isolated from healthy volunteers. Consistent with findings from previous studies, plasma from patients with non-COVID ARDS induced robust NET release by healthy donor neutrophils (Figure 4A). In contrast, plasma from patients with COVID-19 ARDS failed to induce NET release (Figure 4A). There was also no induction of ROS generation in response to plasma from patients with COVID-19 or non-COVID ARDS (Figure 4, B and C). These data demonstrate that functional priming and augmented NET production by neutrophils is not a byproduct of a unique circulating inflammatory milieu (cytokine storm) in this cohort of patients with severe COVID-19.

### Priming of neutrophils in COVID-19 does not alter the response to secondary bacterial challenge.

**Figure 5:**
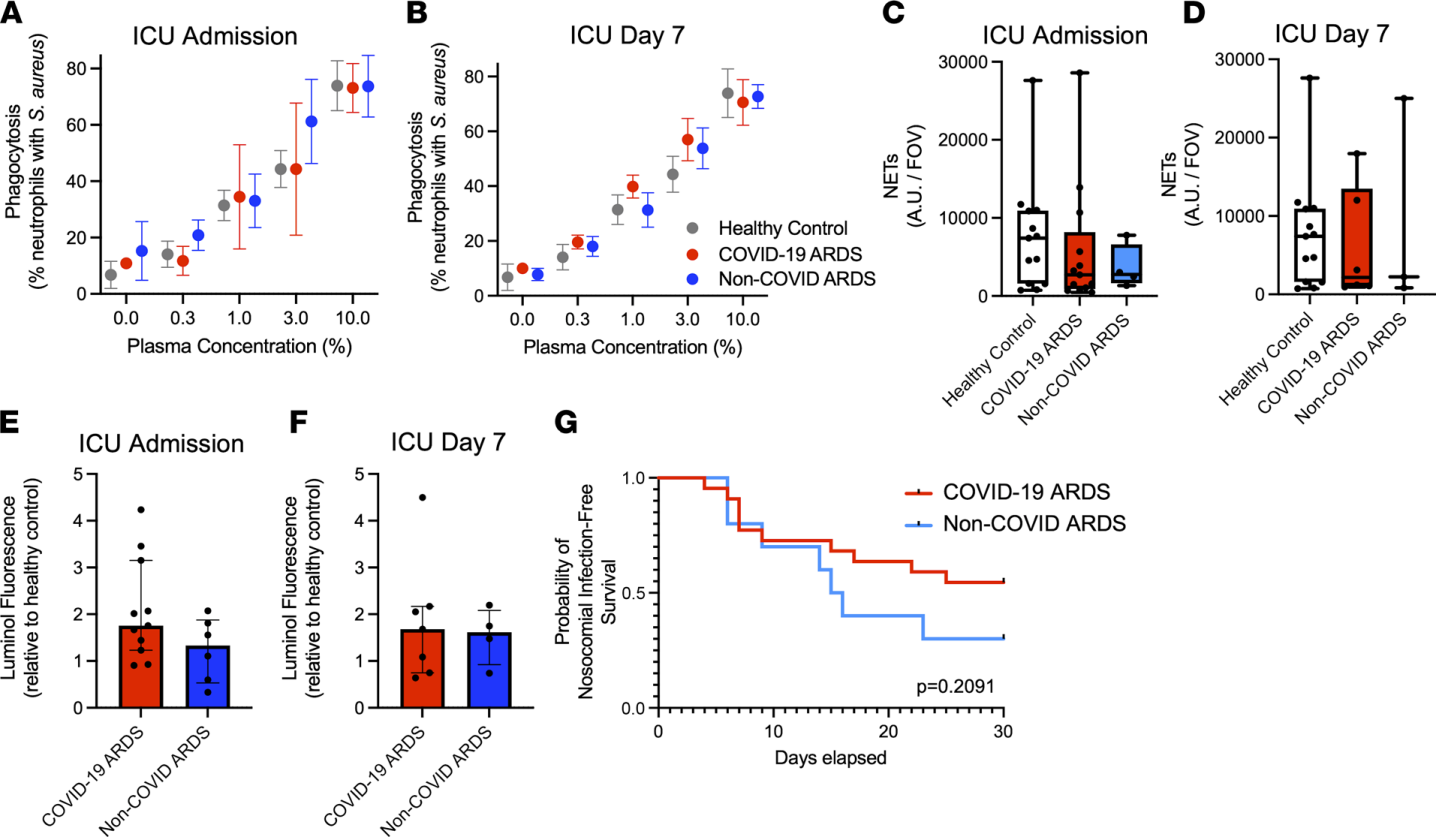
Neutrophils in COVID-19 and non-COVID ARDS remain functionally competent to respond to secondary bacterial challenge. (**A** and **B**) Quantitative assessment of phagocytosis of bacteria (GFP-expressing *S*. *aureus*) by neutrophils using flow cytometry at (**A**) ICU admission and (**B**) day 7 of ICU admission of patients with COVID-19 ARDS and non-COVID ARDS, as well as healthy controls. Data represent the percentage of neutrophils containing GFP^+^ *S*. *aureus*, expressed as mean ± SEM (ICU admission: COVID-19 ARDS, *n* = 5; non-COVID ARDS, *n* = 3; healthy controls, *n* = 5; day 7: COVID-19, *n* = 4; non-COVID, *n* = 3; healthy controls, *n* = 5). (**C** and **D**) Quantification of NET production after stimulation with *S*. *aureus* by neutrophils from patients with COVID-19 (*n* = 13) and non-COVID ARDS (*n* = 4) and healthy controls (*n* = 13) at (**C**) ICU admission and (**D**) day 7 of ICU admission. Dots represent individual patients, bars represent median, and whiskers represent the range. (**E** and **F**) ROS production following stimulation with *S*. *aureus* by neutrophils from patients with COVID-19 ARDS (*n* = 11) and non-COVID ARDS (*n* = 6) detected by luminol fluorescence assay, shown by AUC of luminol fluorescence relative to healthy controls. Dots represent individual patients, bars represent median, and whiskers represent the range. (**G**) Probability of nosocomial infection-free survival of patients with COVID-19 ARDS (*n* = 22) and patients with non-COVID ARDS (*n* = 10) from ICU admission (day 0) to day 30. *P* = 0.2091 by log-rank test.

In addition to their role as pathogenic effector cells in ARDS, dysfunction of neutrophils in critically ill patients also contributes to other complications of ARDS, including high susceptibility to secondary bacterial infections (including ventilator-associated pneumonia) (21). Therefore, we sought to determine if the distinct neutrophil program in COVID-19 ARDS may drive differential susceptibility to secondary bacterial infections compared with non-COVID ARDS. To test this hypothesis, neutrophils were challenged ex vivo with the common nosocomial bacterial pathogen *Staphylococcus aureus* (MRSA, MW2 strain) to determine their ability to mount antibacterial effector mechanisms (phagocytosis, NET production, ROS production) in response to secondary bacterial challenge. Neutrophils isolated from patients with COVID-19 and non-COVID ARDS demonstrated robust capture and phagocytosis of fluorescent *S*. *aureus* (GFP^+^) in both patient groups on admission and after 7 days in ICU, and results were similar in healthy donor neutrophils (Figure 5, A and B). Furthermore, production of NETs (Figure 5, C and D) and ROS (Figure 5, E and F) in response to *S*. *aureus* were also similar between patients with COVID-19 ARDS and patients with non-COVID ARDS. These equivalent effector responses to secondary bacterial challenge are consistent with the finding that nosocomial infections occurred in similar proportions in each patient group, as shown by equivalent nosocomial infection-free survival between patient populations (Figure 5G). This is also consistent with published reports of nosocomial infections in severe COVID-19 (22). Together, these results indicate that neutrophils retain their competence to respond to secondary bacterial pathogens during COVID-19 ARDS, consistent with the observation that differences in clinical outcomes with non-COVID critical ARDS are not driven by differential susceptibility to nosocomial infections (22).

### Pathological neutrophil priming in COVID-19 may escape established therapies, revealing opportunities for targeted therapeutic adjuncts.

**Figure 6:**
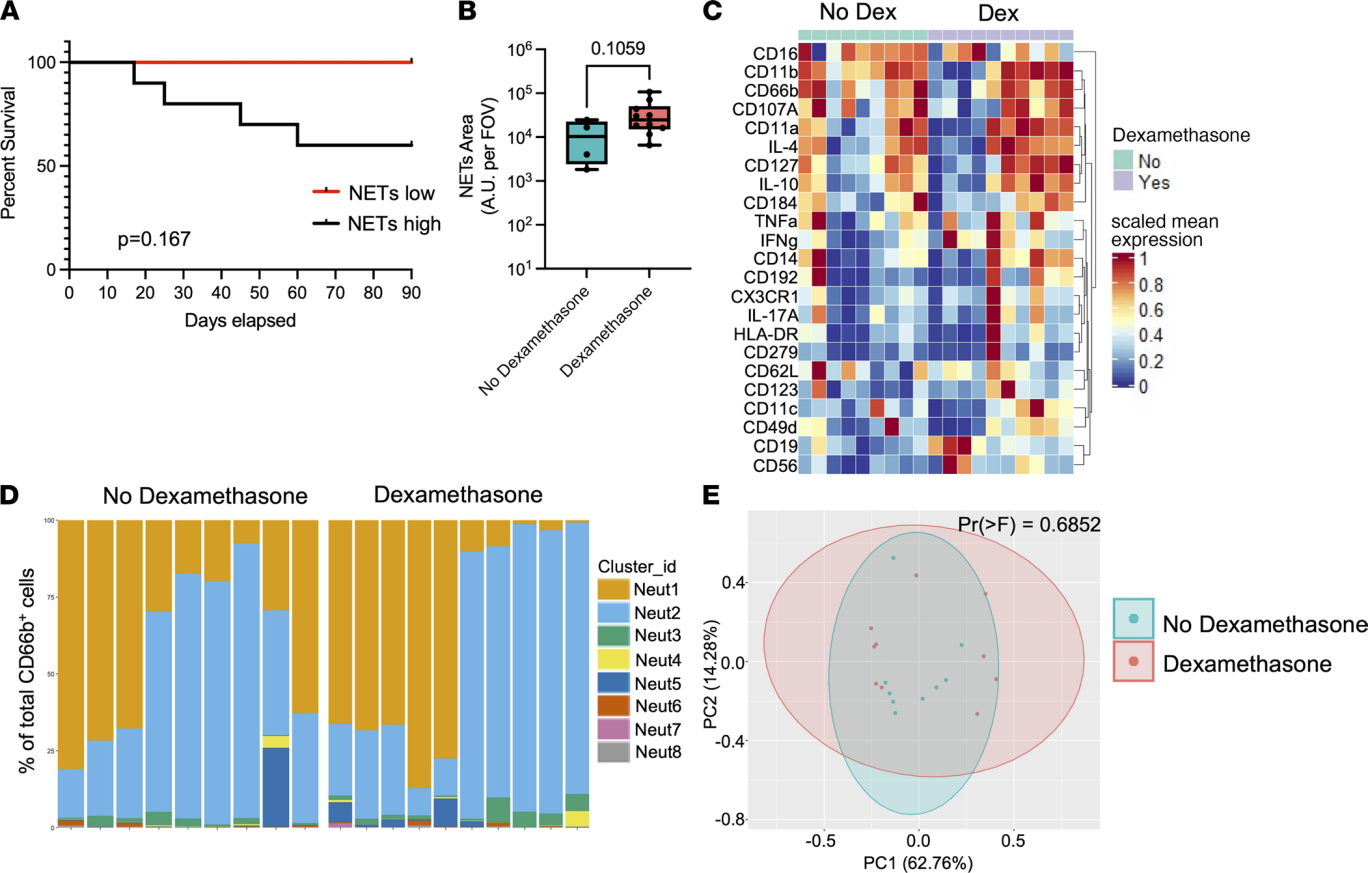
Pathological neutrophil priming in COVID-19 escapes treatment with dexamethasone. (**A**) Probability of survival following admission to ICU (day 0) to 90 days in patients with COVID-19 whose neutrophils produced high (> cohort median) or low (< cohort median) levels of NETs. *P* = 0.167 by log-rank test. (**B**) Quantification of NET production (area per field of covered by NETs) by neutrophils from patients with COVID-19 ARDS who received dexamethasone treatment (*n* = 10) versus those who did not (*n* = 4). Dots represent individual patients at ICU admission, bars represent median, and whiskers represent the range; *P* = 0.1059 by Mann-Whitney *U* test. (**C–E**) Mass cytometry analysis of neutrophils from patients with COVID-19 ARDS who received dexamethasone treatment (*n* = 10) versus those who did not (*n* = 9), showing (**C**) expression levels of selected neutrophil surface and intracellular markers, (**D**) relative abundance of neutrophils clusters in individual patient samples determined by FlowSOM analysis, and (**E**) principal component analysis of neutrophils. *P* = 0.6852 by permutational multivariate ANOVA test.

Emerging data implicate hyperactive NETs as an important mediator of pulmonary vascular and parenchymal injury in severe COVID-19, with recent studies demonstrating an association between surrogate markers of NETs in the circulation and disease severity (16, 17, 23). Given that augmented NET release is a key feature of the distinct neutrophil program observed in our patients with COVID-19, we analyzed the association between NET production and clinical outcomes. Although our sample size was insufficient to achieve statistical significance, a clear trend was observed with 100% 90-day survival in low NET producers (neutrophil NET release < cohort median), compared with only 60% 90-day survival in high NET producers (neutrophil NET release > median of cohort) (Figure 6A). Together with emerging published data, these findings suggest that NETs may be an important pathogenic mediator in COVID-19 and may be amenable to therapeutic targeting (24). Therefore, we aimed to determine whether the clinical efficacy of dexamethasone therapy may be related to its ability to modulate pathological neutrophil priming in COVID-19. We analyzed NET production, surface and intracellular marker expression, and single-cell neutrophil cluster profiles in patients with COVID-19 ARDS who were treated with dexamethasone compared with patients who did not receive dexamethasone (Supplemental Table 1). Surprisingly, we observed no significant difference in NET production nor the phenotypic landscape of circulating neutrophils after treatment with dexamethasone, either early (admission) or later (day 7) during ICU admission (Figure 6, B–E, and Supplemental Figure 5). Given that pathological neutrophil priming and NET production are not modified by dexamethasone treatment, these data suggest that adjunctive therapies (e.g., NET-targeted therapies) may yield additive benefits to dexamethasone and represent an important avenue for further therapeutic development in the fight against COVID-19 ARDS.

## Discussion

Our results add to the growing literature on the importance of neutrophils in the immunopathogenesis of ARDS, including severe COVID-19. It is now well established that patients with SARS-CoV-2 infection display widespread alterations in the myeloid compartment that contribute to disease severity (25, 26). Using high-dimensional single-cell analysis of the entire immune landscape in whole blood, we further this understanding by demonstrating that the systemic immune response in COVID-19 ARDS is dominated by a unique neutrophil compartment characterized by mature and active neutrophil populations. This was in stark contrast to the neutrophil response during non-COVID ARDS, which was much more heterogeneous and enriched with immature neutrophil populations. Further dissecting the functional implications of this unique neutrophil program in COVID-19, we observed that neutrophils from patients with COVID-19 ARDS were functionally primed to release NETs and that high NET producers may be at greater risk of adverse clinical outcomes, consistent with previous reports (17, 23, 24). Surprisingly, this functional priming was not attenuated in patients treated with dexamethasone therapy, thereby revealing this pathological neutrophil program as a potential target for adjunctive therapies to combat COVID-19 ARDS.

We found that neutrophils are functionally primed in COVID-19 ARDS to produce NETs and that this priming was intrinsically programmed during SARS-CoV-2 infection and could not be induced in healthy neutrophils in response to plasma from patients with COVID-19 ARDS. This differed from non-COVID ARDS, as plasma from these patients induced robust NET production by healthy donor neutrophils, consistent with previous studies that have shown NET production in response to plasma from patients with bacterial sepsis and ARDS (19, 20). The cytokine storm of severe SARS-CoV-2 infection has been the focus of extensive investigation to understand the systemic immune drivers of COVID-19. A systematic review and meta-analysis of published cytokine data from 25 studies (totaling 1245 patients) found that substantial differences existed in the levels of core pro- and antiinflammatory cytokines between patients with COVID-19 ARDS and non-COVID ARDS, revealing that levels of key mediators like IL-6 and TNF-α were often much lower in COVID-19 ARDS (18). Furthermore, other recent studies have highlighted that systemic cytokine levels, myeloid cell profiles (including neutrophils), and circulating NETs levels are distinct among patients with COVID-19 who have mild, moderate, and severe disease (4, 16–18). In particular, studies have observed a positive correlation between illness severity and the quantity of NETs in patient plasma. Interestingly, a recent study of patients with mainly mild and moderate COVID-19 found that patient plasma could stimulate NET release from healthy donor neutrophils, which contrasts with our observations in patients with severe COVID-19 ARDS (16). While it is possible that these contrasting results are due to differences in the study cohorts (age, ethnicity, comorbidities), they raise the interesting hypothesis that functional programming and plasticity of neutrophils (including NET release) may be differentially regulated at earlier stages of disease and that early interventions to modulate neutrophil function may help prevent progression to severe disease.

Among the arsenal of neutrophil effector mechanisms, NETs are emerging as important pathological mediators of ARDS in COVID-19 (24). Histopathological analyses of lung tissue from autopsies of patients who have died from COVID-19 have consistently shown that neutrophil infiltration in the lungs is closely associated with NET release, as well as downstream sequelae, including microvascular immunothrombosis (11–14). Quantitative assessment of surrogate markers of NETs in the bloodstream, including DNA-MPO and DNA-citrullinated histone-3 complexes, have suggested that higher NET production is directly correlated with disease severity and may even predict patients who will progress from mild to severe disease (17, 23). This is consistent with our observations that neutrophils from patients with severe COVID-19 are primed to produce NETs and that there was a trend toward higher mortality in patients whose neutrophils produced high levels of NETs. The exact mechanisms that propagate NET production during severe COVID-19 remain an active area of investigation, including modulation of intrinsic pathways of NETosis, direct stimulation and/or infection of neutrophils by SARS-CoV-2 virus, as well as neutrophil-extrinsic mechanisms, including platelet-neutrophil interactions, and autoantibody-mediated stabilization of NETs structures in vivo (12, 14, 27). Interestingly, our finding that augmented NET release was not associated with increased ROS production suggests that the modulation of canonical ROS-dependent NETosis is unlikely to explain our observations. In contrast, our data suggest that the neutrophil pool in patients with severe COVID-19 is enriched with more mature and functionally active subsets that are intrinsically primed to release NETs, including a trend toward higher NET production in patients whose neutrophil pool was enriched with Neut2 subset, further underpinning the potential importance of a programmed neutrophil response toward the unique pathogenesis of COVID-19.

The findings of this study lend further support to the potential therapeutic utility of targeting pathological neutrophil mechanisms, including NETs, for the treatment of COVID-19 ARDS (24). In particular, we found that dexamethasone treatment did not modulate the functional landscape of neutrophils or attenuate the production of NETs. This is in line with a publication suggesting that dexamethasone does not alter the NET-related neutrophil proteome in patients with severe COVID-19 (28). Of note, our findings do not rule out the possibility that dexamethasone modulates other aspects of neutrophil development or activation in vivo, as has been suggested using transcriptomic analysis of the circulating neutrophil pool in severe COVID-19 (29). Together, these data suggest that neutrophil-mediated pathogenesis, including augmented NET production, may escape the treatment effect of dexamethasone and, therefore, represent a promising avenue for therapeutic adjuncts to supplement current treatment regimens.

Finally, this study uncovers a number of outstanding questions regarding the role of neutrophils in the pathogenesis of COVID-19 and ARDS. First, our study focuses on the immune landscape in the bloodstream compartment, but understanding the implications of our findings within the lung microenvironment may reveal important features of tissue-specific neutrophil responses in COVID-19. A recent study of the single-cell landscape of lung immunity in SARS-CoV-2 infection, including spatially resolved profiling with imaging mass cytometry, observed a distinct pattern of neutrophil infiltration in the lungs of patients with severe COVID-19 compared with non-COVID pneumonia (9). However, much remains to be learned about whether there is selective recruitment of specific neutrophil populations to the lungs in COVID-19 (e.g., NET-producing neutrophils), and the molecular mechanisms of neutrophil recruitment within the pulmonary microvasculature need to be defined. Indeed, it was recently reported that neutrophil recruitment to the lungs in ARDS utilizes a unique adhesion molecule (DPEP-1), which is now being targeting in clinical trials for moderate-to-severe COVID-19 (ClinicalTrials.gov NCT04402957) (30). In addition, outstanding questions remain about the molecular mechanisms that program the systemic neutrophil landscape in COVID-19, and further research is needed to define the mechanisms that elicit pathological NET release within lung tissues. Much remains to be learned about the mechanisms driving differences between neutrophil programming in COVID and non-COVID (primarily bacterial) pneumonia and ARDS, such as the contribution of specific viral versus bacterial products, differential systemic inflammatory responses, and tissue-level contributions within various neutrophil niches (bone marrow, blood, and lung). Finally, our modest sample size was necessary to enable deep profiling of neutrophil phenotypes and functions but limits our ability to address their effects on clinical outcomes. Although we did observe an association between neutrophil priming and high NET production with mortality in COVID-19 ARDS, a larger study will be required to confirm the impact of our findings on adverse clinical outcomes in severe COVID-19 (degree of hypoxemia, illness severity, multiorgan dysfunction, thrombotic complications, and death). However, the demonstration of a functionally distinct neutrophil landscape in severe COVID-19 that escapes conventional therapy with dexamethasone provides further support for the ongoing efforts to develop neutrophil/NET-targeted therapies to treat COVID-19 ARDS.

## Methods

### Study design.

Between April 1, 2020, and March 30, 2021, consecutive patients were screened for the following inclusion criteria: adult patients (>18 years of age), with an index admission to 1 of 4 multisystem ICUs in Calgary, Alberta, Canada, with a diagnosis of ARDS based on the Berlin criteria (31), associated with a diagnosis of SARS-CoV-2 infection (based on clinical laboratory qPCR assay from nasopharyngeal swab or endotracheal tube aspirate) or bacterial pneumonia (based on standard clinical and microbiological criteria). Exclusion criteria included preexisting immunocompromised state (immunomodulatory therapy, chemotherapy, HIV infection, or other congenital or acquired immunodeficiency), readmission to ICU or prior study enrollment, ARDS due to another cause, goals of care that excluded life-support interventions, or moribund patients not expected to survive more than 72 hours. Blood samples were collected from enrolled patients with COVID-19 (*n* = 22) and those with non-COVID (*n* = 10) ARDS on admission to ICU and day 7 (for those who survived and remained in ICU). As this was an observational study, patient management was directed exclusively by the treating physicians and was not affected by our study. Patients with COVID-19 who received dexamethasone therapy were treated as per the RECOVERY trial protocol with 6 mg dexamethasone daily for 10 days (15), and the median time from initiation of treatment to study enrollment was 2 days (range 0–8 days). Clinical outcomes of nosocomial infection were identified using a standard definition (32) of new infections occurring at least 48 hours after admission, diagnosed by the treating clinicians, that resulted in administration of new antimicrobial treatment. Blood samples were also collected from healthy volunteers for use as controls.

### TOF mass cytometry and analysis.

Whole blood samples used for mass cytometry analysis were cryopreserved in PROT1 proteomic stabilizer (SmartTube) at a ratio of 1:1.4 and stored at –80°C to enable batched analysis of patient samples as previously described (33). Samples were thawed at room temperature, followed by red blood cell lysis using PROT1 red blood cell lysis buffer (SmartTube). White blood cells were washed in cell staining medium (PBS with 1% BSA) followed by labeling with a custom metal-conjugated antibody panel (Supplemental Table 2). First, cells were incubated with metal-conjugated surface antibodies, followed by fixation and permeabilized (BD Cytofix-Cytoperm) and incubation with intracellular antibodies. Finally, cells were incubated overnight in a solution containing Cell-ID iridium intercalator (Fluidigm), 0.3% saponin, and 1.6% paraformaldehyde in PBS. Cells were then mixed with EQ Four Element Calibration Beads (Fluidigm) and acquired on a Helios CyTOFII mass cytometer (DVS). Mass cytometry data were normalized using the internal Helios CyTOFII bead-based normalization software (DVS).

For mass cytometry data analysis, FCS files were imported into Cytobank (http://premium.cytobank.org) for data visualization and manual gating. Single-cell events were then exported from CytoBank as FCS files and loaded into the CATALYST package (34) in R (R Development Core Team). For neutrophil clustering and analysis, CD45^+^CD66b^+^ neutrophils were analyzed by FlowSOM (35), and a Consensus Clustering method (36) was employed on all neutrophil single-cell events within CATALYST based on the expression of neutrophil markers (see Figure 1C). To determine the optimal number of metaclusters, we compared the change in the AUC of the CDF plot for each additional metacluster added (*delta_area* function; CATALYST). Using this method, we identified 11 neutrophil metaclusters. Very rare metaclusters (representing less than 0.5% of total neutrophils) were excluded from the analysis, leaving 8 metaclusters in the analysis. To visualize the neutrophil metacluster landscape, tSNE dimensionality reduction was performed on 2500 randomly selected events from each sample using a perplexity of 70 for 5000 iterations. Data visualization was generated using the built-in functions of CATALYST and the ggplot2 package in R.

### Plasma and neutrophil isolation.

Blood samples collected in heparinized tubes were centrifuged at 450*g* and pelleted blood cells were resuspended in PBS. Neutrophils were isolated using a 2-density histopaque gradient (1.119 g/mL and 1.077 g/mL; MilliporeSigma, 11191 and 10771, respectively) with centrifugation at 400*g* for 20 minutes at 20°C without braking. The layer of neutrophils (second layer) was collected, red blood cells were lysed, and isolated neutrophils were resuspended in RPMI containing 0.05% of human serum albumin at a final concentration of 1 × 10^7^ neutrophils/mL. Neutrophil viability was more than 98%, as determine by trypan blue staining. Plasma samples were generated by centrifugation of blood samples at 2000*g* for 10 minutes at 4°C. Of note, not all patient samples yielded sufficient quantities of neutrophils and plasma to conduct every assay, but every effort was made to include as many patients as possible in all assays.

### NET release assay.

Isolated neutrophils (5 × 10^4^) were seeded into sterile 96-well optical plates (Falcon) and allowed to settle for 5 minutes at room temperature. The cells were incubated under the following conditions: unstimulated, 0.1 μM PMA, or 1 × 10^6^ CFU of *S*. *aureus* MW2 for 3 hours at 37°C with 5% CO_2_. To assess NET release in response to patient plasma, the isolated neutrophils from healthy volunteers were incubated with 5% patient plasma containing 5 mM EDTA for 3 hours at 37°C with 5% CO_2_. Cells were then fixed with 2% paraformaldehyde overnight and then stained with Sytox Orange (Thermo Fisher Scientific) to visualize extracellular DNA NETs and imaged on an inverted spinning disk confocal microscope with a 10× objective. The quantity of NET release was determined by measuring that visualized area covered by extracellular DNA NETs per field of view.

### ROS production assay.

Isolated neutrophils (1 × 10^6^) were incubated with luminol (50 μM) in the presence of superoxide dismutase (75 μg/ml), catalase (2000 U/ml), and horseradish peroxidase (20 U/ml), in an opaque 96-well plate. In some experiments, neutrophils were coincubated with *S*. *aureus* MW2 (1 × 10^6^ CFU). Chemiluminescence was read using a Spectramax i3x instrument (Molecular Devices) every 1 minute for 120 minutes.

### Phagocytosis assay.

Methicillin-resistant *S*. *aureus* MW2, constitutively expressing EGFP, was grown overnight in Brain Heart Infusion broth (Thermo Fisher Scientific) supplemented with 10 μg/mL chloramphenicol. On the day of the experiment, the strain was subcultured to late log phase (OD_660nm_ 1.0), washed, and resuspended in RPMI-containing 0.05% human serum albumin. Subsequently, 2 × 10^5^ isolated neutrophils were mixed with 1 × 10^6^ bacteria (MOI = 5) in the presence of various concentrations of normal human pooled serum (15 donors) in a final volume of 200 μL RPMI containing 0.05% HAS, and incubated at 37°C with agitation for 15 minutes. The cells were then fixed with 4% paraformaldehyde, and phagocytosis was measured using flow cytometry (FACS Canto). Neutrophils were gated based on their forward and side scatter profiles, and bacterial phagocytosis was measured as the percentage of neutrophils positive for GFP^+^ bacteria using FlowJo software (FloJo, BD Biosciences).

### MPO-DNA complex and cfDNA quantification.

cfDNA was quantified in plasma samples using the Quanti-iT PicoGreen kit (Invitrogen) according to the manufacture’s instructions, and concentrations were calculated based on a standard curve of λ DNA (Invitrogen). MPO-DNA complex levels were quantified by ELISA using a protocol modified from (37). Briefly, a 96-well flat-bottom plate was coated with anti-human MPO antibody (Bio-Rad) overnight at 4°C and then washed 4 times with PBS-tween buffer before blocking with PBS/4% BSA. The samples were then incubated with patient plasma for 2 hours at room temperature. After 4 washes with PBS-tween, the quantity of DNA bound to captured MPO was quantified using the Quant-iT PicoGreen kit (Invitrogen) as per the manufacturer’s instructions.

### Statistics.

Nonparametric data are represented as median ± range or interquartile range and were analyzed using Mann-Whitney *U* test (when comparing 2 groups) or Kruskal-Wallis test with post hoc Dunn’s test for multiple comparisons (when comparing more than 2 groups). Parametric data are represented with mean ± SEM and were analyzed using a 2-tailed Student’s *t* test (when comparing 2 groups) or 1-way ANOVA with post hoc Bonferroni correction for multiple comparisons when more than 2 groups were compared. Principal component analysis of neutrophil marker expression between patient groups was performed using the *procomp* function and VEGAN package in R and plotted using ggplot2. Permutational multivariate ANOVA analysis of the Bray-Curtis distances between individual samples was used to compare principal component analysis ordinations. A *P* value less than 0.05 was considered significant. Statistical analysis and graphic generation were performed using Graphpad Prism and R (R Development Core Team).

### Study approval.

This study was approved by the conjoint health research ethics board of the University of Calgary and Alberta Health Services (REB18-1294, 20-0481). Written informed consent was obtained from all study participants or appropriate surrogate decision makers for patients who were unable to provide consent.

## Author contributions

RP, FVSC, JMS, HBS, BGJS, and BM contributed to the design of the study, data acquisition, and data analysis. AZZ made contributions to data acquisition and data analysis. ZS, BGY, and BGJM contributed to patient enrollment, sample collection and processing, and clinical data acquisition. PK contributed to the design of the study and data analysis. RP, FVSC, JMS, and BM wrote the manuscript, and all authors contributed to the editing process and approved the manuscript. Authorship order was determined by relative contribution.

## Supplementary Material

Supplemental data

## Figures and Tables

**Table 1:**
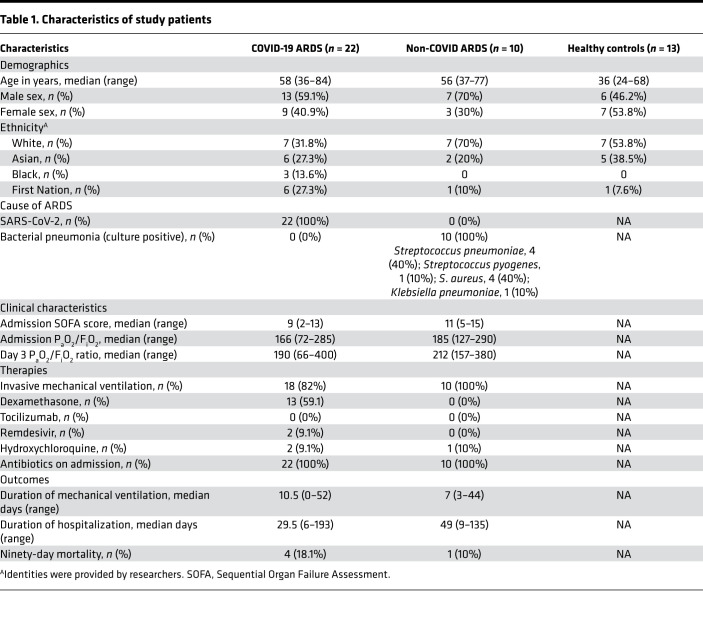
Characteristics of study patients

## Version 1 12/15/2021

In-Press Preview

## Version 2 01/25/2022

Electronic publication
