# Supplementary material for: A functionally distinct neutrophil landscape in severe COVID-19 reveals opportunities for adjunctive therapies
Source: JCI Insight. 2022 Jan 25;7(2):e152291. doi: 10.1172/jci.insight.152291 (PMC8855826; doi:10.1172/jci.insight.152291)
Supplement: Supplemental data [file jciinsight-7-152291-s047.pdf]

Supplementary Data for

A functionally distinct neutrophil landscape in severe COVID-19 reveals opportunities for adjunctive therapies

Rachita Panda*[1,2], Fernanda VS Castanheira*[1,2], Jared Schlechte*[1,3], Bas GJ Surewaard[1,4], Hanjoo Brian Shim[1,2], Amanda Z Zucoloto[1,3], Zdenka Slavikova[3], Bryan G Yipp[1,3], Paul Kubes[1,2,3], Braedon McDonald[1,3]

[1]Snyder Institute for Chronic Diseases, Cummings School of Medicine, University of Calgary, Calgary, AB, Canada
[2]Department of Physiology and Pharmacology, Cummings School of Medicine, University of Calgary, Calgary, AB, Canada
[3]Department of Critical Care Medicine, Cummings School of Medicine, University of Calgary, Calgary, AB, Canada
[4]Department of Microbiology, Immunology, and Infectious Diseases, Cummings School of Medicine, University of Calgary, Calgary, AB, Canada
*These authors contributed equally to this work.

Supplementary Figure 1

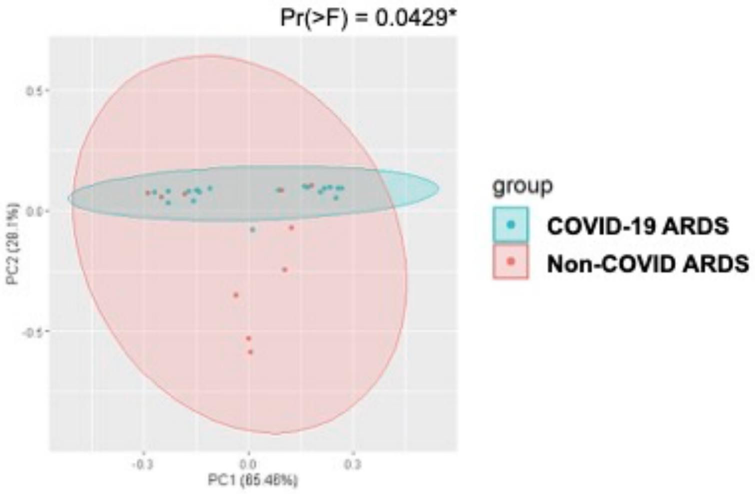

Supplementary Figure 1. Principal component analysis of neutrophil cluster abundance (from Figure 2C) at ICU admission in patients with COVID -19 ARDS (N=19) and Non-COVID ARDS (N=10). *p<0.05 by PERMANOVA test.

Supplementary Figure 2

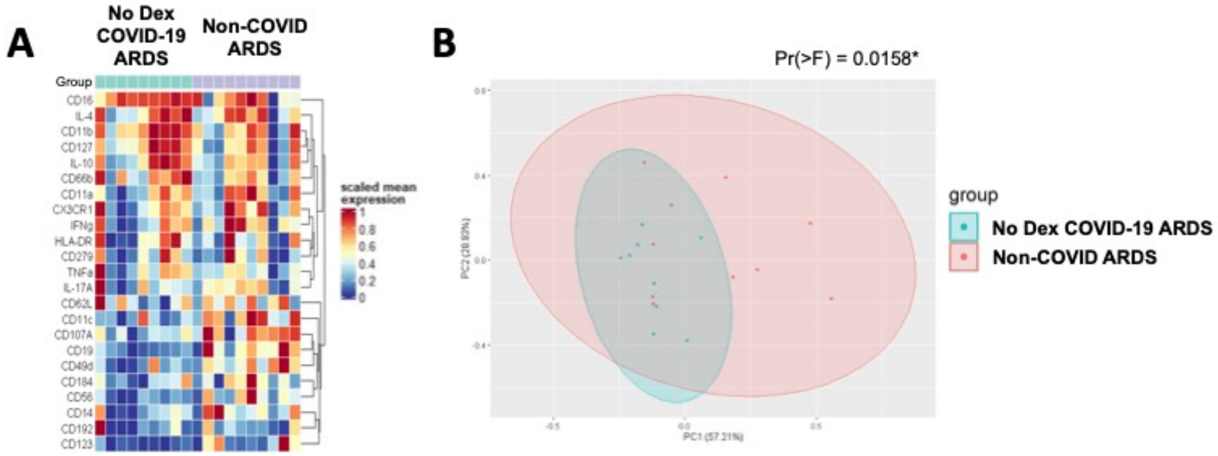

Supplementary Figure 2. Subgroup analysis of comparing (A) expression levels of neutrophil surface and intracellular markers, and (B) principal component analysis of neutrophil marker expression at ICU admission in patients with non-COVID ARDS (N=10) versus patients with COVID-19 ARDS who did not receive dexamethasone treatment (N=9). *p<0.05 by PERMANOVA test.

Supplementary Figure 3

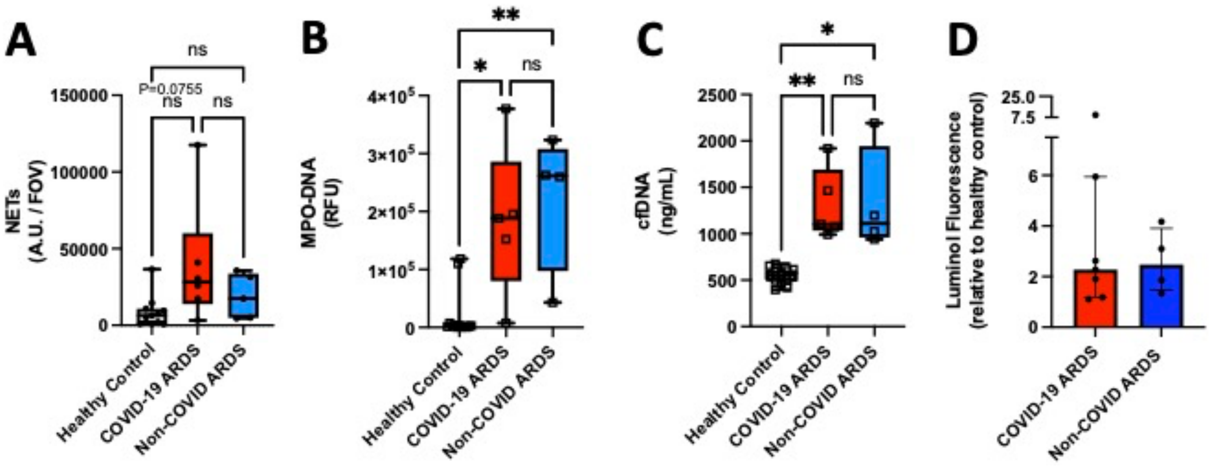

Supplementary Figure 3. NETs and ROS production by neutrophils on day 7 of ICU admission. (A) Quantitation of area per field of view covered by NETs released from neutrophils ex vivo, as well as plasma concentrations of (B) MPO-DNA complexes, and (C) cell-free (cf) DNA in patients with COVID-19 (N=5-6) and non-COVID ARDS (N=4-5) at ICU day 7 as well as healthy controls (N=11-13). Data are individual patients, with median and range, $^{*}p<0.05$ $^{**}p<0.01$ by Kruskal-Wallis test with post-hoc Dunn's test. (D) Reactive oxygen species (ROS) production by neutrophils detected by luminol fluorescence assay shown as AUC in patients with COVID-19 ARDS (N=7) and non-COVID ARDS (N=4) relative to healthy control neutrophils. Data are individual patients, with median and interquartile range.

Supplementary Figure 4

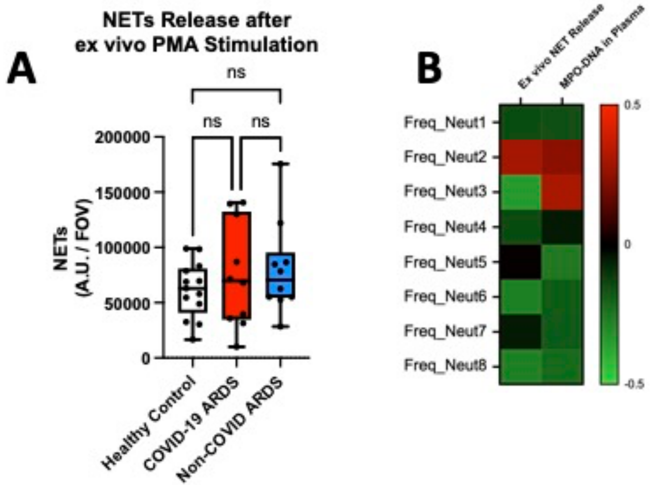

Supplementary Figure 4. (A) Quantification of NETs production (area per field of view covered by NETs) following stimulation with PMA by neutrophils from healthy controls (n= 13), COVID-19 ARDS (n= 10) and non-COVID ARDS (n=10). Data are individual patients, with median and range, ns = non-significant by Kruskal-Wallis test with post-hoc Dunn's test. (B) Heatmap depicting the Spearman correlation coefficient between the frequency (relative abundance) of individual neutrophil clusters versus the quantity of NETs measured by ex vivo NETs release or plasma MPO-DNA complexes.

Supplementary Figure 5

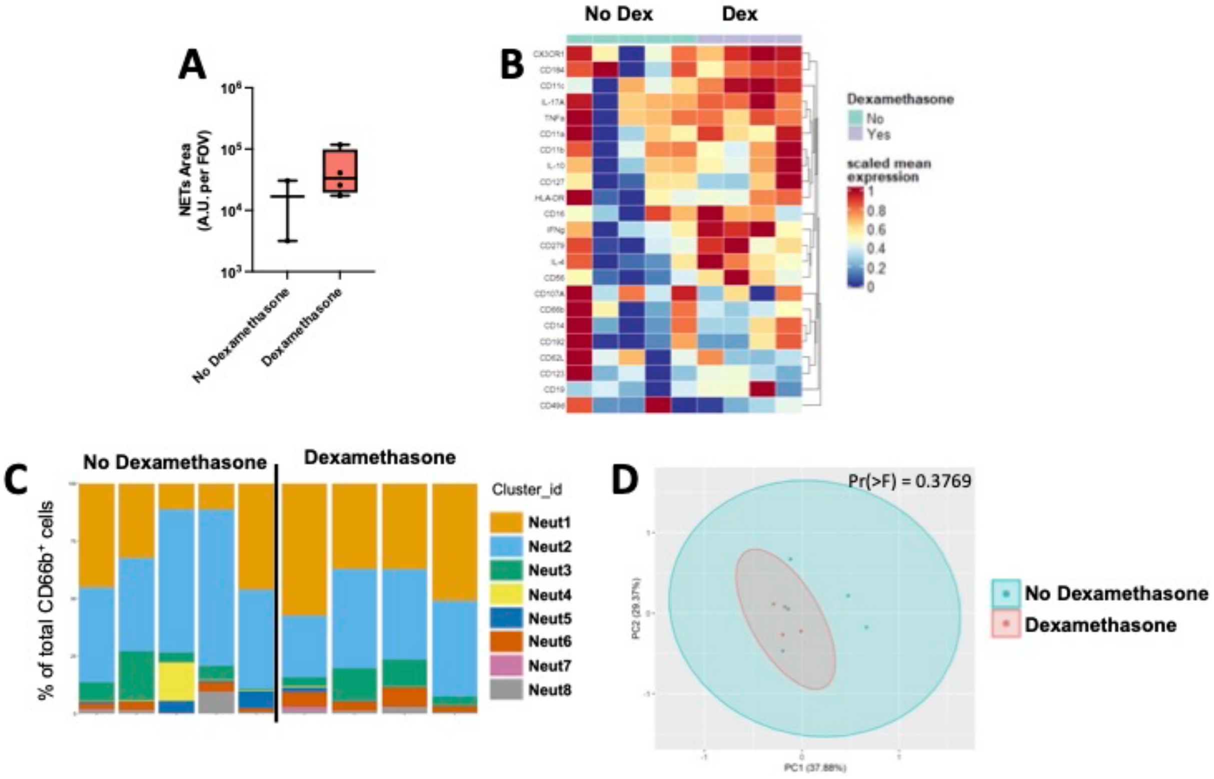

Supplementary Figure 5. Pathological neutrophil priming in COVID-19 escapes treatment with dexamethasone on ICU day 7. (A) Quantification of NETs production (NETs area per field of view) by neutrophils from patients with COVID-19 ARDS who received dexamethasone treatment (N=4) versus those who did not (N=2). Data are individual patients on ICU day 7 of ICU admission, with median and range. (B-D) Mass cytometry analysis of neutrophils from patients with COVID-19 ARDS on ICU day 7 who received dexamethasone treatment (N=4) versus those who did not (N=5), demonstrating (B) expression levels of selected neutrophil surface and intracellular markers, (C) relative abundance of neutrophils clusters in individual patient samples determined by FlowSOM analysis, and (D) principal component analysis of neutrophils, p=0.3769 by PERMANOVA test.

Supplementary Table 1

Supplementary Table 1 - Patient characteristics for COVID-19 patients managed with or without dexamethasone therapy

**Characteristics**	**Dexamethasone (n=13)**	**No Dexamethasone (n=9)**
Demographics		
Age - median (range)	62 (36 - 84)	56 (45 - 73)
Male sex - n (%)<br>Female sex - n (%)	7 (53.8%)<br>6 (46.2%)	6 (66.7%)<br>3 (33.3%)
Ethnicity<br>White<br>Asian<br>Black<br>Indigenous	 3 (23.1%)<br>3 (23.1%)<br>3 (23.1%)<br>4 (30.8%)	 4 (44.4%)<br>3 (33.3%)<br>0<br>2 (22.2%)
Cause of ARDS		
SARS-CoV-2	100%	100%
Clinical Characteristics		
Admission SOFA Score - median (range)	7 (2 - 13)	10 (1 - 11)
Admission P_aO_2/F_iO_2 ratio - median (range)	172 (72 - 270)	145 (102-285)
Day 3 P_aO_2/F_iO_2 ratio - median (range)	203 (66 - 314)	168 (83 - 400)
Therapies		
Invasive mechanical ventilation - n (%)	11 (84.6%)	7 (77.8%)
Dexamethasone - n (%)	13 (100%)	0 (0%)
Tocilizumab - n (%)	0 (0%)	0 (0%)
Remdesivir - n (%)	2 (15.4%)	0 (0%)
Hydroxychloroquine - n (%)	0 (0%)	2 (22.2%)
Antibiotics on admission - n (%)	13 (100%)	9 (100%)
Outcomes		
Duration of mechanical ventilation - median days (range)	10 (0 - 44)	11.5 (0 - 52)
Duration of hospitalization - median days (range)	21 (6 - 62)	50 (8 - 193)
90 day mortality - n (%)	3 (23.1%)	1 (11.1%)

Supplementary Table 2

Supplementary Table 2 - Mass cytometry antibody panel

Marker	Clone	Metal	Source
CD45	HI30	89Y	Fluidigm
CD3	UCHT1	141Pr	Fluidigm
CD19	HIB19	142Nd	Fluidigm
CD107A	H4A3	143Nd	Biolegend*
CD4	RPA-T4	145Nd	Fluidigm
CD8a	RPA-T8	146Nd	Fluidigm
CD11c	Bu15	147Sm	Fluidigm
IL-17A	BL168	148Nd	Fluidigm
CD56	NCAM16.2	149Sm	Fluidigm
CD192	M-T701	150Nd	Biolegend*
CD123	6H6	151Eu	Fluidigm
TNFα	Mab11	152Sm	Fluidigm
CD62L	DREG-56	153Eu	Fluidigm
CD49d	9F10	154Sm	Biolegend*
CD45RA	HI100	155Gd	Fluidigm
CD184	12G5	156Gd	Fluidigm
IFNγ	B27	158Gd	Fluidigm
FoxP3	259D/C7	159Tb	Fluidigm
CD14	M5E2	160Gd	Fluidigm
CD66b	80H3	162Dy	Fluidigm
IL-4	MP4-25D2	163Dy	Fluidigm
Granulysin	RB1	165Ho	Biolegend*
IL-10	JES3-9D7	166Er	Fluidigm
CD11b	ICRF44	167Er	Fluidigm
CD127	A019D5	168Er	Fluidigm
CD25	2A3	169Tm	Fluidigm
CD11a	HI111	170Er	BIolegend*
Granzyme B	GB11	171Yb	Fluidigm
CX3CR1	2A9-1	172Yb	Fluidigm
HLA-DR	L243	173Yb	Fluidigm
CD279	EH12.2H7	174Yb	Fluidigm
Perforin	BD48	175Lu	Fluidigm
CD7	K036C2	176Yb	Biolegend*
CD16	3G8	209Bi	Fluidigm
Cell-ID Ir	n/a	Ir191 and Ir193	Fluidigm

*metal conjugation performed in house
